# *Spiroplasma* infection in *Harmonia axyridis* - Diversity and multiple infection

**DOI:** 10.1371/journal.pone.0198190

**Published:** 2018-05-29

**Authors:** Irina Goryacheva, Alla Blekhman, Boris Andrianov, Denis Romanov, Ilya Zakharov

**Affiliations:** 1 Vavilov Institute of General Genetics, Russian Academy of Sciences, Moscow, Russia; 2 Koltsov Institute of Developmental Biology, Russian Academy of Sciences, Moscow, Russia; University of Innsbruck, AUSTRIA

## Abstract

The heritable endosymbiotic bacterium *Spiroplasma* is found in the harlequin ladybird *Harmonia axyridis*. The proportion of beetles infected with *Spiroplasma* in different native *H*. *axyridis* populations varies from 2% to 49%. We investigated the polymorphism of *Spiroplasma* strains in samples from individual beetles from Kyoto, Vladivostok, Troitsa Bay, Novosibirsk, and Gorno-Altaisk. To identify *Spiroplasma* strains, we analyzed nucleotide polymorphisms of the 16S rRNA gene and the ribosomal internal transcribed spacer (ITS1). The majority of infected beetles were infected with two or more *Spiroplasma* strains. We measured *Spiroplasma* density in beetles with different infection status using quantitative PCR. The abundance of *Spiroplasma* in samples with a single infection is an order of magnitude lower than in samples with multiple infections. Density dependent biological effects of *Spiroplasma* are discussed.

## Introduction

Many insect species are infected with intracellular symbiotic bacteria, which are inherited maternally and affect host reproduction. Among such bacteria, *Wolbachia*, *Spiroplasma*, *Rickettsia*, and *Cardinium* are widely known. *Spiroplasma* is one of the most prevalent and well characterized facultative insect endosymbionts, and it is estimated to infect 5–10% of all insect species [1, 2]. This bacterium is an endosymbiont of some members of all the main insect orders: Coleoptera [3–8], Diptera [1, 9–15], Hemiptera [16–18], Homoptera [19–22], Lepidoptera [23, 24], and Odonata [25]. *Spiroplasma* is found in the midgut, filter chamber, malpighian tubules, hindgut, fat tissues, hemocytes, muscle, trachea, salivary glands, reproductive tissues and eggs [23, 26, 27]. Phylogenetic reconstruction of the genus *Spiroplasma* using the 16S rRNA gene sequences revealed that the genus comprises four distinct clades: the *Ixodetis* clade, the Citri-Chrysopicola-Mirum clade, the Apis clade sensu *lato*, and the Mycoides-Entomoplasmataceae clade [28, 29].

In insects, the effects of symbiosis with *Spiroplasma* range from mutualistic to parasitic. In some insects, *Spiroplasma* determines male-killing at an embryogenesis stage [5, 8, 30], resulting in strongly female-biased offspring. The male-killing spiroplasmas are known in *Drosophila* [31, 32], ladybird beetles: *Adalia bipunctata*, *Anisosticta novemdecimpunctata*, *Harmonia axyridis*, *Menochilius sexmaculatus* [3, 5, 6, 8, 33]; in lepidopterans: *Danaus chrysippus*, *Ostrinia zaguliaevi* [23, 24], and in Oriental tea tortrix (*Homona magnanima)* [34]. Male-killing *Spiroplasmas* from *Drosophila* belong to the Citri-Chrysopicola-Mirum clade [15]. The male-killing effect for *Spiroplasma* strains in their native host is density-dependent [35]. Strains that do not exhibit male-killing effect have a lower *Spiroplasma* density at all stages of the host’s life cycle compared with the male-killing *Spiroplasma* strains. The highest density at all stages of the *Drosophila* life cycle was noted for male-killing *Spiroplasma* which infects *Drosophila melanogaster*. A strain that causes male-killing in *D*. *melanogaster* (its native host) also does so in *Drosophila neotestacea*, even though these insects diverged 40–60 mya, indicating that male-killing is not strongly dependent on host genetic background [36]. In *H*. *axyridis*, *Spiroplasma* induces early male-killing phenomena in some Japanese populations revealed by genetic and cytological analysis [3], and much later *Spiroplasma* infection of Japanese and continental populations of *H*. *axyridis* was confirmed by molecular-genetic methods [6, 33]. This *Spiroplasma* belongs to the Ixodetis clade. Japanese populations of *H*. *axyridis* are infected with two strains of *Spiroplasma*, named HARFUKU1 and HARFUKU2 [37]. The proportion of females infected with *Spiroplasma* in different populations was estimated to be 49% in Sapporo [38], 4% in Muikamachi and 14% in Fukuyama [37], about 10% in Western Siberia [33], and about 2% in the Altai [38]. In all these populations, there was a shift in the sex ratio toward females. In addition, to male-killing, *Spiroplasma* also affects other biological characteristics of infected *H*. *axyridis* females, such as reduced adult female survival and reduced female embryo survival [39, 40]; reduced development time, with the strongest effect seen at the larval stage; and increased body size of infected insects [41]. Greater body size in *Spiroplasma*-infected *H*. *axyridis* corresponds to a greater ovariole number [41]. Data on the effect of *Sprioplasma* on the *H*. *axyridis* fertility are ambiguous. Some authors report a reduced fertility of females infected with *Spiroplasma* [39, 40], while others suggest an increase in potential fertility [39]. Shortening the developmental time, increasing body size and fecundity in *Spiroplasma* infected *H*. *axyridis*, and the effect on the sexual structure of the population may have important implications for the *H*. *axyridis* biology [41].

The aim of this work was to investigate the polymorphism of intracellular *Spiroplasma* bacteria in Western and Eastern populations of *H*. *axyridis* and to study the relative density of different bacterial strains in one host with single and multiple infections. A significant proportion of infected females are characterized by multiple (e.g. double, triple) infections. The density of a bacterial population in insects with multiple infections is an order of magnitude higher than the density of a bacterial population in insects infected by a single *Spiroplasma* strain. We discuss the potential consequences of multiple *Spiroplasma* infections.

## Materials and methods

### DNA sampling

To study *Spiroplasma* polymorphism from *H*. *axyridis*, we used DNA samples of 16 *Spiroplasma*-infected beetles from Eastern and Western populations, of which the infectious status was determined previously by us [42]. Twelve samples originate from females from the Eastern population, including six samples from Vladivostok (V) and Troitsa Bay (Tr) and two samples from Kyoto (K), as well as four samples from females of the Western population, including two samples from Novosibirsk (N) and two samples from Gorno-Altaisk (G). Map of *H*. *axyridis* sampling locations in S1 Fig. In two *H*. *axyridis* samples from Novosibirsk, two new *Spiroplasma* strains (GenBank ID: KR363169, KR363170) were detected previously by us [42]. *H*. *axyridis* is not a protected insect species. Collections of beetles were conducted in the territories of municipalities that do not limit the collection of *H*. *axyridis*. No specific permissions were required for collecting *H*. *axyridis* in these territories.

Ethical approval is not required for this study because we use only DNA preparations and do not experiment in any way with living insects.

### Amplification

All DNA samples from beetles were tested before use. To validate the prepared templates, the mitochondrial cytochrome oxidase 1 (*COI*) gene region of *H*. *axyridis* was amplified by PCR primers LCO1490 and HCO2198 [43]. To detect *Spiroplasma* infection and to study *Spiroplasma* diversity, we used *Spiroplasma*-specific primers that amplify the 301-bp DNA fragment including ITS1 complete sequence [6], and *Spiroplasma*-specific primers that amplify the 1028 bp fragment of 16S rRNA gene [18] (Table 1). The amplification reaction was conducted under the following conditions: initial denaturation (one cycle 4 min at 95°С), followed by 38 cycles that include denaturation (30 s at 95°С), annealing (40 s at 59°С for ITS1 and 40 s at 53°С for gene fragment of 16S rRNA), and polymerization 40 s at 72°С. This was followed by a cycle of final polymerization (5 min at 72°С). For a positive control, we used a DNA sample of *H*. *axyridis*, infected by *Spiropasma* from the DNA collection at the Vavilov Institute of General Genetics RAS. For a negative control, we used DNA samples from uninfected *H*. *axyridis* imago from the same collection.

**Table 1:** Primers used in this work for amplification of *Spiroplasma* and *H*. *axyridis* sequences.

PCR fragment name	Fragment length (bp)	Primer name	Primers sequence	Primers melting temperature (Tm).	Reference
Fragments of the rRNA repeat of *Spiroplasma sp*					
ITS1	301	SP-ITS-JO4	5'-GCCAGAAGTCAGTGTCCTAACCG-3'	Tm = 59°C	[6]
		SP-ITS-N55	5'-ATTCCAAGCCATCCACCATACG-3'	Tm = 59°C	
16S rRNA	1028	spi_f1	5'-GGGTGAGTAACACGTATCT-3'	Tm = 53°C	[17]
		spi_r3	5'-CCTTCCTCTAGCTTACACTA-3'	Tm = 53°C	
Fragments of the nuclear gene of *H*. *axyridis*					
carbamoylphosphate synthetase (CPS)	711	CPS-F	5'-TGGCCAGTAAAGCAACTGGT-3'	Tm = 60°C	This paper
		CPS-R	5'-CCATCACATGTTCCTCATCAA-3'	Tm = 59° C	

### qPCR

Using a NanoDrop8000 (Termo Scientific, Germany), DNA concentrations were measured. Samples were then diluted as necessary so that each were of the same DNA concentration for qPCR. Real-time qPCR reactions were carried out in the ANK-32 real-time PCR system (Syntol, Russia). Quantitative PCR was performed in 25-μL reactions containing Lightcycler 480 SYBR Green I (Invitrogen) and 0.5 μM of each of the primers. The following thermal cycling protocol was applied: 95°C for 5 min followed by 35 cycles at 95°C for 10 s, 60°C for 10 s, then 72°C for 30 sec. Three technical replicates per biological sample were performed for each set of primers, SP-ITS-JO4 and SP-ITS-N55 for the internal transcribed spacer (ITS1) of *Spiroplasma* and CPS-F and CPS-R for the carbamoyl phosphate synthetase (CPS) gene of *H*. *axyridis* (Table 1). Melting curves were examined to confirm the specificity of amplified products. Cycle threshold (Ct) values were obtained using the ANK-32 real-time PCR system at default threshold settings. The efficiency of each primer pair was predetermined in separate experiments using serial 10-fold dilutions of the DNA samples. The amplification efficiency of CFP (E = 1.94) is different from that of the *Spiroplasma* ITS1 sequence (E = 1.72). Therefore, qPCR from CFP was used to confirm *H*. *axyridis* total DNA quantity (Table 2). To estimate differences in *Spiroplasma* DNA quantities between samples, ratios of *Spiroplasma* DNA quantities between samples were calculated as: No/Mo = E^ΔCt^, where (No) is the initial concentration in sample N, (Mo) is the initial concentration in sample M, and (ΔCt) is the difference in the number of control cycles between samples N and M. The quantity of *Spiroplasma* ITS1 DNA in sample N19 was used as a proxy for *Spiroplasma* DNA titers in other samples. To determine the 95% confidence interval of the mean Ct, the one-sample Student t test was used. To compare relative quantifications of *Spiroplasma* DNA in samples with single and multiple infections, the Mann-Whitney test was used.

**Table 2:** Measurement of *H*. *axyridis* samples DNA concentration.

№	Beetle number	Identified *Spiroplasma* strains	DNA concentration (ng/ul)	Ct FAM (CFP gene of *H*. *axyridis*) Three technical PCR replicates	Mean number of Ct FAM (CFP gene of *H*. *axyridis*) with 95% confidence interval
1	G9	HARFUKU2	1.2	26.33; 26.91; 26.62	26.62 ± 0.72
2	G13	HARFUKU2	1.2	26.05; 26.88; 26.72	26.55 ± 1.09
3	N1	Sib1	1.2	25.80; 26.01; 26.12	26.21 ± 0.64
4	N19	Sib19	1.2	26.38; 26.98; 26.11	26.49 ± 1.11
5	G 16	HARFUKU1; HARFUKU2	1.2	26.50; 26.10; 25.79	26.13 ± 0.88
6	V37	HARFUKU1; HARFUKU2; Bi24	1.2	26.89; 26.55; 27.32	26.92 ± 0.96
7	V42	HARFUKU2; Bi10	1.2	27.19; 26.15; 26.67	26.67 ± 1.29
8	T5	HARFUKU1; HARFUKU2; Bi22; Tr54; Tr55	1.2	26.11; 26.55; 26.00	26.22 ± 0.72
9	Т21	HARFUKU1; HARFUKU2; Tr21	1.2	26.32; 25.95; 26.69	26.32 ± 0.92
10	Bi16	HARFUKU1; HARFUKU2; Bi24; Bi10	1.2	26.60; 26.53; 27.06	26.73 ± 0.72
11	Bi29	HARFUKU1; HARFUKU2; Bi24; Bi22	1.2	27.84; 26.54; 26.14	26.84 ± 2.21
12	K11	HARFUKU1; HARFUKU2	1.2	25.94; 26.85; 26.17	26.32 ± 1.18
13	K15	HARFUKU1; HARFUKU2	1.2	26.72; 26.78; 27.20	26.90 ± 0.65

Ct FAM–The value of the reference cycle (fluorescent dye: SYBR Green I).

### Electrophoresis, elution, cloning, and sequencing

PCR products were run on an 1.5% agarose gel, then extracted from the gel and purified with an elution kit (Zymoclean™ Gel DNA Recovery Kit, Zymo Research, USA), according to the manufacturer’s instructions. PCR product cloning was performed using the pGEM®-T Easy Vector System, according to standard protocols (Fermentas InsTAclone™ PCR Cloning Kit). We use random sampling of the clones for sequencing. Sequencing of the amplification products was conducted with both primers on an ABI PRISM 3500 instrument using a BigDye® Terminator v3.1 Cycle Sequencing Kit (Applied Biosystems, United States), according to the manufacturer’s instructions.

### Multiple sequence alignment and phylogenetic analysis

Sequences were aligned using the Clustal W algorithm in the MEGA 4.00 program package [44]. As a reference sequence for the rRNA gene cluster, we used *Spiroplasma* sequence from the closely related *H*. *axyridis* species of ladybirds *Anisosticta novemdecimpunctata* (GenBank ID: AM087471). The Median-Joining network of the *Spiroplasma* rRNA genes from different strains was constructed in the PopART program [45] using the TCS algorithm [46].

## Results

*Spiroplasma* ITS1 and the fragment of 16S rRNA gene nucleotide variability are presented in Tables 3 and 4. *Spiroplasma* sequences from females G9, K13, N19, and N1 showed no evidence of ambiguous nucleotide positions. This allowed us to conclude that these females were infected with a single *Spiroplasma* strain. The ITS1 sequences from G9 and K13 females were identical to the sequence of the *Spiroplasma* strain HARFUKU2 that was previously found in *H*. *axyridis* from Japan (GenBank ID: AB127933) [37]. The ITS1 sequences in samples N1 and N19 were previously recorded as the Sib1 strain (GenBank ID: KR363169) and Sib19 strain (GenBank ID: KR363170), respectively.

**Table 3:** *Spiroplasma* ITS1 nucleotide variability. Nucleotides of phylogenetically informative polymorphic positions are indicated.

Names of *H*. *axyridis* infected females	Names of *Spiroplasma* strains	Nucleotide positions**														
		1 4 8 4	1 4 9 7	1 5 3 5	1 5 3 8	1 5 4 7	1 5 6 2	1 5 6 8	1 5 6 9	1 5 7 3	1 5 8 5	1 5 8 8	1 5 9 2	1 5 9 8	1 6 2 8	1 6 6 0
	HARFUKU1*	**T**	**G**	**A**		**G**	**T**	**G**	**A**	**T**	**C**	**C**	**T**	**G**	**C**	**C**
	HARFUKU2 *	**C**				**A**					**A**					**T**
G9; K13;	HARFUKU2*	**C**				**A**					**A**					**T**
N19	Sib19	**C**														
N1	Sib1	**G**	**C**	**G**	**T**		**C**	**T**	**G**	**C**	**A**	**A**		**C**	**T**	
G16; Bi16, Bi29; T5, T21; V37, V42; K11, K15		**Y**				**R**					**M**					**Y**

*according to [37]

** As a reference sequence we used ribosomal genes cluster of *Spiroplasma* from *A*. *novemdecimpunctata* (GenBank ID: AM087471). The letters indicate the collection site: Bi–Birobidzhan, G–Gorno-Altaisk, K–Kyoto, N–Novosibirsk, T–Troitsa Bay, V–Vladivostok. The characters indicate the sample numbers.

**Table 4:** Nucleotide variability of 16S rRNA fragments of *Spiroplasma* of *H*. *axyridis*. Nucleotides of phylogenetically informative polymorphic positions are indicated.

Names of *H*. *axyridis* infected females				Nucleotide positions**															
	1 7 7	1 8 2	1 8 7	2 1 6	2 3 2	2 6 2	2 6 5	2 6 6	2 6 9	4 4 4	4 4 5	4 5 8	4 6 6	6 1 7	6 2 3	6 9 5	8 9 0	9 8 3	1 0 5 1
Reference strain -*Spiroplasma* from *A*. *novemdecimpunctata*	G	A	G	N	A	T	C	G	G	G	A	T	C	C	G	G	C	G	G
N19		**G**		**G**															
N1	**A**		**A**	**G**	**G**	**C**	**T**	**T**		**T**	**C**	**G**	**T**	**T**	**A**		**T**	**A**	**C**
G9; K13;				**A**												**A**			
G16; Bi16, Bi29; T5, T21; V37, V42; K11, K15				**R**					**K**							**R**			

** The numbers of the variable nucleotides are given by the sequence of the ribosomal genes cluster of *A*. *novemdecimpunctata* (GenBank ID: AM087471), which was used as a reference sequence.

However, in most *Spiroplasma*-infected *H*. *axyridis* females from Gorno-Altaisk (G16), Birobidzhan (Bi16, Bi29), Troitsa Bay (T5, T21), Vladivostok (V37, V42), and Kyoto (K11, K15), some nucleotides were ambiguously read at phylogenetically informative sites of both ITS1 and in the fragment of 16S rRNA gene. Because the emergence of ambiguous sites could indicate multiple infections in females, we cloned PCR fragments and then a set of individual clones were sequenced. We cloned the ITS1 fragment with ambiguous sites of all samples and the 16S gene fragment of three samples: G16, V37 and Tr21.

*Spiroplasma* strain identification of individual clones is presented in Tables 5 and 6. Newly obtained sequences of the *Spiroplasma* 16S rRNA gene and ITS1 were deposited into GenBank (GenBank ID: KR363166-KR363168, MF543310-MF543312). Multiple infections with *Spiroplasma* strains were detected in *H*. *axyridis* females from five studied populations in both (western and eastern) parts of the native range. HARFUKU1 and HARFUKU2 *Spiroplasma* strains co-occur in most females with multiple infections. HARFUKU1 strain is absent only in one female from Vladivostok (V42, Table 5). Rare *Spiroplasma* strains were detected in multiply infected females from Birobidzhan, Vladivostok and Troitsa Bay. One beetle from Japan (K13) and one beetle from the Gorno-Altaisk (G9) are infected only by HARFUKU2. The sequences of the Sib19 strain and Bi22 strain found in females from Troitsa Bay and Birobidzhan were identical.

**Table 5:** ITS1 based identification of *Spiroplasma* strains from individual females of *H*. *axyridis*.

Female number of *H*. *axyridis*	Strain name (number of clones in parenthesis)	GenBank ID:
**Western populations**		
G16	(1)–HARFUKU1	AB127932
	(5)–HARFUKU2	AB127933
G9	HARFUKU2	AB127933
N1	Sib1	KR363169
N19	Sib19	KR363170
**Eastern populations**		
Bi16	(2)–HARFUKU1	AB127932
	(14)–HARFUKU2	AB127933
	(2)–Bi24	KR363168
	(1)–Bi10	KR363166
Bi29	(3)–HARFUKU1	AB127932
	(11)–HARFUKU2	AB127933
	(1)–Bi24	KR363168
	(2)–Bi22	KR363167
V37	(7)–HARFUKU1	AB127932
	(6)–HARFUKU2	AB127933
	(1)–Bi24	KR363168
V42	(14)–HARFUKU2	AB127933
	(1)–Bi10	KR363166
T5	(6)–HARFUKU1	AB127932
	(6)–HARFUKU2	AB127933
	(4)–Bi22	KR363167
	(4)–Tr54	MF543310
	(2)–Tr55	MF543311
T21	(6)–HARFUKU1	AB127932
	(10)–HARFUKU2	AB127933
	(3)–Tr21	MF543312
K11	(2)–HARFUKU1	AB127932
	(12)–HARFUKU2	AB127933
K15	(5)–HARFUKU1	AB127932
	(9)–HARFUKU2	AB127933
K13	HARFUKU2	AB127933

Note: the numbers in parentheses indicate the number of plasmid clones of this type in a random sample set of clones.

**Table 6:** 16S rRNA based identification of *Spiroplasma* strains from individual females of *H*. *axyridis*.

Female number of *H*. *axyridis* and geographical locations	Strain name (number of clones in parenthesis)	GenBank ID:
**Western populations**		
G16	(8) Ha1	MF538703
	(7) Ha2	MF538704
G9;	Ha2	MF538704
N1	Ha4	MG672513
N19	Ha5	MG672514
**Eastern populations**		
V37	(8) Ha1	MF538703
	(7) Ha2	MF538704
	(2) Ha3	MF538705
Tr21	(2) Ha1	MF538703
	(13) Ha2	MF538704
K13	Ha2	MF538704

Note: the numbers in parentheses indicate the number of plasmid clones of this type in a random sample set of clones.

Because HARFUKU1 and HARFUKU2 have the same length and differ only by few point nucleotide substitutions that do not alter the structure of the PCR fragments ends, we assume that possible differences in the amplification efficiency of these fragments in one reaction and possible differences in the cloning efficiency are negligible. Therefore, we approximate the quantitative relationship of *Spiroplasma* strains in the case of multiple infection from the number of plasmid clones of different types in a random sampling of clones (Tables 5 and 6). In a random sampling of clones, we found a total of 26 HARFUKU1 and 87 HARFUKU2 strains. Based on this data, we propose that the density of HARFUKU2 may be more than twice the density of HARFUKU1 in beetles with double infection. Analysis of the 16S rRNA gene polymorphism confirms the presence of multiple infections in females. Overall, we identified 45 clones of this fragment: 27 clones corresponding to the Ha-2 strain and 18 clones corresponding to the Ha-1 strain. One beetle from Japan (K13) and one beetle from the Altai (G9) are infected only by Ha-2 strain. Since it is known that the ITS1 sequence is physically linked to the 16S rRNA sequence in the ribosomal gene cluster and based on the infection of the beetles K13 and G9 with only one *Spiroplasma* strain it can be assumed that Ha2 16S rRNA fragment is physically linked to HARFUKU2 fragment of ITS1. Consequently Ha1 16S rRNA fragment is linked to HARFUKU1 fragment of ITS1. Quantitative data of the number of clones of different types supports this assumption. Obviously, the На3 sequence of the 16S rRNA fragment found in the V37 female, is linked with the Bi24 ITS1 strain and Ha4 and Ha5 sequences of the 16S rRNA fragment found in females from Novosibirsk infected with a single *Spiroplasma* strain, are linked with Sib1 and Sib19 fragments of ITS1 respectively.

### Phylogenetic analysis

**Fig 1:**
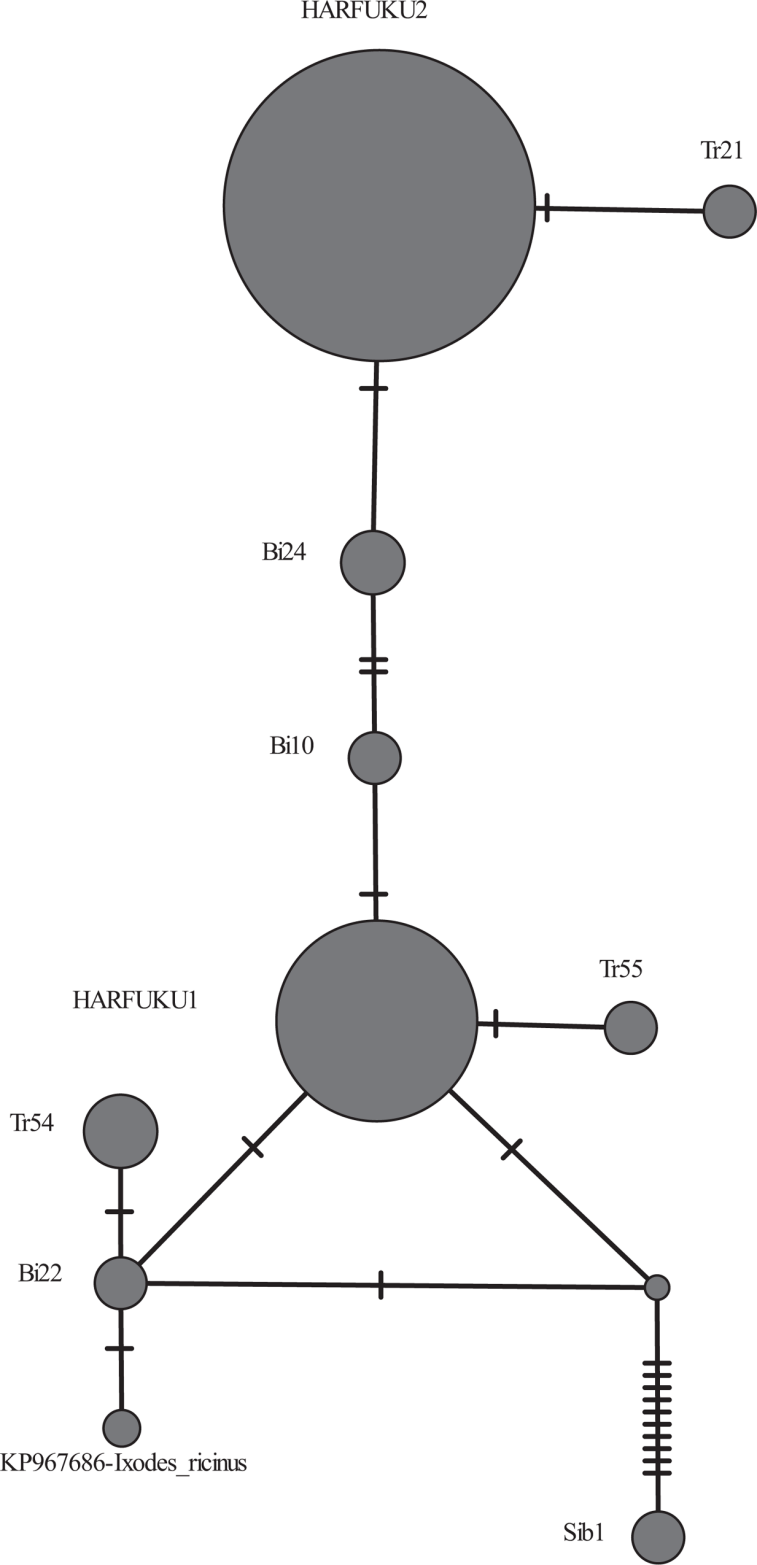
ITS1 median network of *Spiroplasma* strains from *H*. *axyridis*. A reconstruction based on the analysis of the internal transcribed spacer ITS1 polymorphism. ITS1 strain sequences are shown in Table 5. Mutations are indicated by dashes. The size of the circles is proportional to the number of sequences in each group. *Spiroplasma* from *Ixodites ricinus* was used as a control.

**Fig 2:**
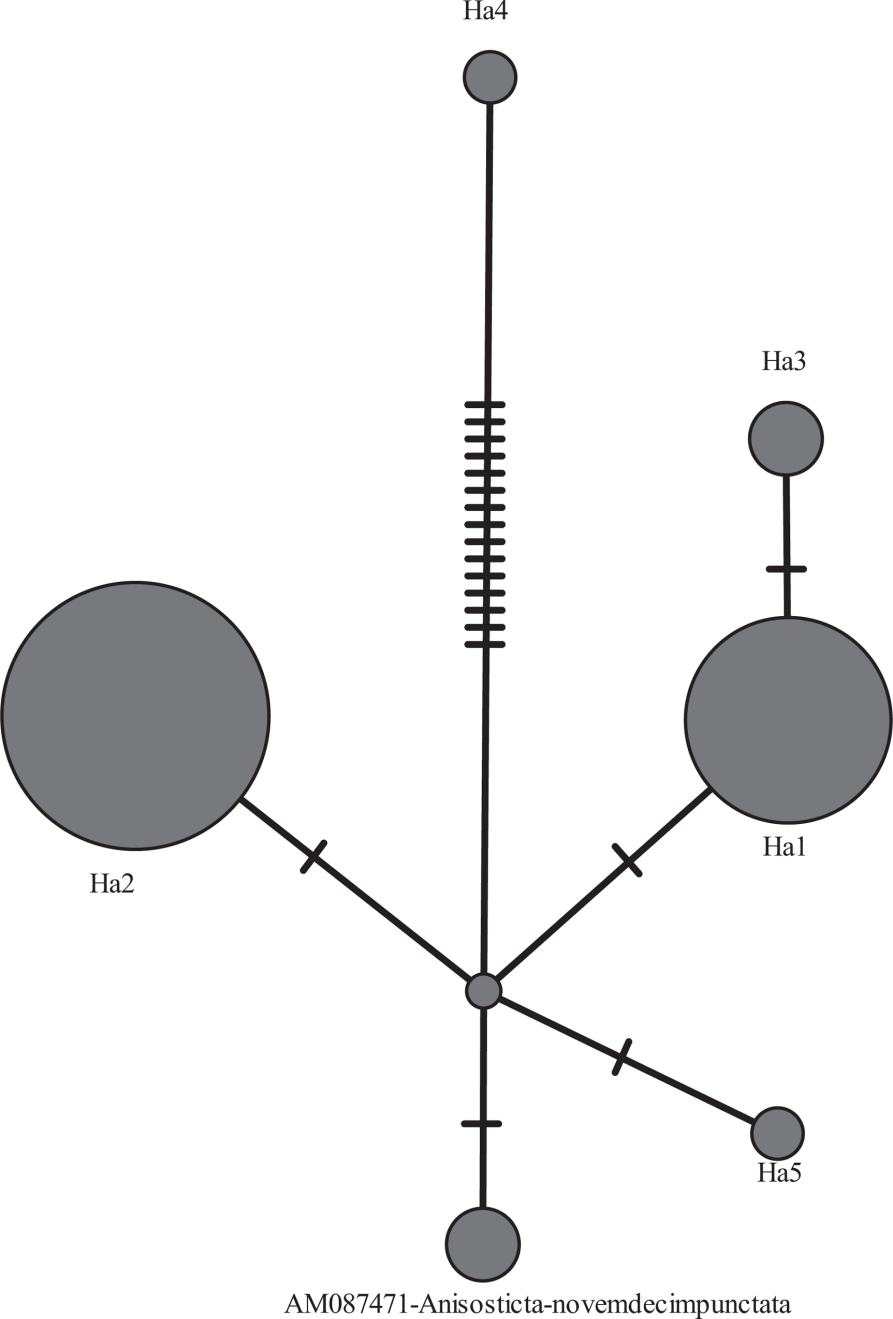
16S rRNA median network of *Spiroplasma* strains from *H*. *axyridis*. This reconstruction is based on the analysis of 16S rRNA gene fragment polymorphisms. Characteristics of the strains are presented in Table 6. Mutations are indicated by dashes. The size of the circles is proportional to the number of sequences in each group. *Spiroplasma* from *A*. *novemdecimpunctata*, a closely related ladybird species, was used as a control.

The results of the phylogenetic analysis of ITS1 and the 16S rRNA gene fragment of *Spiroplasma* from *H*. *axyridis* are presented in Figs 1 and 2. All identified *Spiroplasma* strains belong to the Ixodetis clade. The nucleotide divergence of the ITS1 fragment is π = 0.0091. There are 17 variable sites, and of these, there are 16 phylogenetically informative sites. The nucleotide divergence of the 16S rRNA fragment is π = 0.002. There are 19 variable sites, four of them are phylogenetically informative.

### Survey for abundance of *Spiroplasma* in *H*. *axyridis*

The *Spiroplasma* relative densitys were measured in 13 DNA samples from *H*. *axyridis*. The results are shown in Table 7. To verify the data we divided our sampling into two groups. The first group included four beetles (G9, G13, N1, N19) infected with only one *Spiroplasma* strain. The second group included beetles with multiple infections. Comparison of the groups based on the value of *Spiroplasma* relative densities was performed using the Mann-Whitney test. The value of Mann–Whitney U test (U-empirical = 0. Is less then U-critical = 3 for p≤0.01) confirms that this groups are different. We may conclude that the relative density of *Spiroplasma* in samples with a single infection is lower than in samples with multiple infections. All samples with high *Spiroplasma* density are infected simultaneously with strains HARFUKU1 and HARFUKU2. This is likely to be one of the necessary conditions to achieve high *Spiroplasma* density.

**Table 7:** Relative density of *Spiroplasma* in females with single and multiple infections.

№	Beatle number	Identified *Spiroplasma* strains	DNA concentration (ng/ul)	Ct FAM (ITS1 *Spiroplasma*) of three technical PCR replicates	Mean number of Ct FAM (ITS1 *Spiroplasma*) with 95% confidence interval	*Spiroplasma* density *
**1**	**G9**	**HARFUKU2**	**1.2**	32.44; 31.90; 31.27	31.87 ± 1.45	**1.53**
**2**	**G13**	**HARFUKU2**	**1.2**	31.39; 31.90; 32.50	31.93 ± 1.39	**1.49**
**3**	**N1**	**Sib1**	**1.2**	33.05; 32.40; 31.00	32.15 ± 2.60	**2.27**
**4**	**N19**	**Sib19**	**1.2**	31.95; 33.00; 33.03	32.66 ± 1.53	**1.00**
5	G 16	HARFUKU1; HARFUKU2	1.2	24.92; 24.60; 24.49	24.67± 0.55	76.18
6	V37	HARFUKU1; HARFUKU2; Bi24	1.2	25.22; 25.12; 23.79	24.71 ± 1.98	74.55
7	V42	HARFUKU2; Bi10	1.2	26.22; 26.33; 26.47	26.34 ± 0.31	30.80
8	T5	HARFUKU1; HARFUKU2; Bi22; Tr54; Tr55	1.2	25.02; 24.57; 23.91	24.50 ± 1.39	83.54
9	Т21	HARFUKU1; HARFUKU2; Tr21	1.2	23.61; 25.34; 23.05	24.00 ± 2.97	109.56
10	Bi16	HARFUKU1; HARFUKU2; Bi24; Bi10	1.2	23.07; 23.89; 25.40	24.12 ± 2.94	102.66
11	Bi29	HARFUKU1; HARFUKU2; Bi24; Bi22	1.2	23.22; 24.43; 24.11	23.92 ± 1.56	114.42
12	K11	HARFUKU1; HARFUKU2	1.2	24.22; 23.92; 23.26	23.80 ± 1.22	122.12
13	K15	HARFUKU1; HARFUKU2	1.2	24.22; 24.11; 23.04	23.79 ± 1.62	122.78

* The *Spiroplasma* density in sample N19 was used as a proxy for *Spiroplasma* titers.

Ct FAM–The value of the reference cycle (fluorescent dye: SYBR Green I).

## Discussion

We detected nine *Spiroplasma* strains within populations of the *H*. *axyridis* native range. The two most common strains–HARFUKU1 and HARFUKU2 –as well as two minor strains–Sib1 and Sib19 –have been previously noted in Japanese and Novosibirsk populations, respectively [42, 37]; the rest of the strains described here are recorded for the first time. Our study is the first to note the diversity of *Spiroplasma* from a single host. Previously, the diversity of *Spiroplasma* strains was investigated in the genus *Drosophila*. In Drosophilidae, the infected species has a single *Spiroplasma* strain [15]. However, a diversity of strains that infect one host species have been repeatedly discussed for other reproductive endosymbiotic bacteria. In particular, the diversity of *Wolbachia* strains was found in *Drosophiala simulans*, *Drosophila melanogaster*, *Culex pipiens* [47, 48, 49]; a diversity of *Rickettsia* and *Arsenophonus* strains were found in *Bemisia tabaci* invasive biotype [50]. Interpretation of the data of *Rickettsia* and *Arsenophonus* diversity in *B*. *tabaci* requires some caution; it is known that *B*. *tabaci* is a cryptic species complex comprising at least 24 morphologically indistinguishable species [51].

All *Spiroplasma* strains previously detected in *H*. *axyridis* belong to the Ixodetis clade. The Sib1 strain belongs to the *Spiroplasma* cluster whose members infect arachnids; three other strains are phylogenetically close to the male-killing *Spiroplasma* from *A*. *bipunctata* [42]. Five new *Spiroplasma* lines (Fig 2), which differ from previously identified strains by single nucleotide substitutions, also belong to the Ixodetis clade and are phylogenetically close to *Spiroplasma* from the ladybird beetle *Anistotica novemdecempunctata*. The position on the median network (Fig 1) of the Bi22 strain, which is closely related to the *Spiroplasma* strain from *Ixodites ricinus*, suggests that this strain is ancestral to the remaining *Spiroplasma* strains of *H*. *axyridis*, with the exception of the Sib1 strain. The remaining strains can be considered to be derivatives of the primary ones via the accumulation of point mutations. However, on the other hand, the HARFUKU2 strain could be acquired independently by *H*. *axyridis*. In this case, the Bi10 and Bi24 strains could arise as a result of recombination between HARFUKU1 and HARFUKU2, which is possible when the strains coexist in one individual. Such coexistence has been demonstrated in our study. The formation of new lines via the recombination of the original lines coexisting in the same organism was previously noted in *Wolbachia* [52–57]. However, evidence of *Spiroplasma* strain recombination requires an analysis of a longer genome fragment than we had in our possession. The Sib1 strain, found only in one female from the Novosibirsk population, likely infected *H*. *axyridis* independently. The uniqueness of the *Spiroplasma* Sib1 strain infecting female N1 from Novosiborsk is supported by data pertaining to the variability of both ITS1 and 16S rDNA (Figs 1 and 2).

The *Spiroplasma* strains detected in this study exist in various combinations and quantitative ratios in different females and in different populations of the native range of *H*. *axyridis*. The proportion of HARFUKU2 (Ha2) (Tables 5 and 6) in the total strain pool is more than 62%. This strain is found in females with both multiple and single infections (Table 5). The HARFUKU1 strain is also widespread in the *H*. *axyridis* area, where it is detected at a frequency of nearly 22%. Although we found this strain only in multiply infected females in combination with the strain HARFUKU2, in Japanese populations HARFUKU1 occurs in females with a single infection [42]. The Bi22 strain, which is identical to the Sib19 strain (5% in the total strain pool) was detected in *H*. *axyridis* as a single infection only in Novosibirsk (as Sib19), and as a multiple infection in females from the eastern part of the range (Table 5). Minor strains of Tr21, Tr55 and Tr54 were found only in two females with multiple infections in the Troitsa Bay population. The Bi10 and Bi24 strains were detected in four females from two different populations of the eastern part of the range.

Single-strain *Spiroplasma* female infections were detected only in Novosibirsk (Sib1, Sib19), Gorno-Altaisk (HARFUKU2), and Kyoto (HARFUKU2) populations, which are all located on the periphery of the native range. In the same edge populations (Gorno-Altaisk and Kyoto), the diversity of *Spiroplasma* strains was reduced in multiply infected females, which carry only two *Spiroplasma* strains (HARFUKU1 and HARFUKU2). The number of *Spiroplasma* strains ranged from 3–5 in multiply infected females from other populations (Table 5). We hypothesize that a decrease in the overall diversity of *Spiroplasma* strains from the center to the periphery of the range, both in one host individual and in populations as a whole, reflects the well-known pattern of the microevolutionary process, which consists of reductions in diversity in edge populations [58–60]. The possibility cannot be excluded that *Spiroplasma* reduces the fitness of infected beetles in conditions of ecological pessimum at the border of the *H*. *axyridis* native range (Novosibirsk, Gorno-Altaisk). In this case, a mono infection and a decrease in bacterial abundance may be considered to be a stage of *Spiroplasma* elimination from the populations of *H*. *axyridis*. The assumption of the negative *Spiroplasm*a effect on the viability of *H*. *axyridis* is indirectly supported by the absence (or very low occurrence) of *Spiroplasma* in invasive populations of *H*. *axyridis* [61].

The diversity of *Spiroplasma* strains in *H*. *axyridis* is the result of at least two events of infection of the host. The existence of repeated infections of *H*. *axyridis* was previously shown for its symbiotic bacteria *Rickettsia* [61] and *Wolbachia* [62]. Multiple infection events are known for other symbiotic pairs. *D*. *simulans* is infected by five strains of *Wolbachia* that span across both supergroup A and B, including three supergroup A strains and two supergroup B strains [47, 63]. These strains attained a different density in the host cells and, accordingly, determined different levels of cytoplasmic incompatibility, as well as different levels of protection against pathogens.

In most of the samples studied, we detected infection with two or more *Spiroplasma* strains. The same type of infection, more than a single strain in one host–can be assumed in ticks. At least in the study of the symbiotic community structure in *Zygiella x-notata* Clerck, 1757 (Araneae: Araneidae) within the *Spiroplasma*, there was evidence for several “heterozygous nucleotide positions”. Individual *Spiroplasma* ITS sequences contained up to eight such sites [64], which we suggest is the result of multiple infections. Multiple infections are also known for *Wolbachia* [65–67]. Because multiple infections are widespread among *Spiroplasma* infected individuals (88%), it is possible that such co-infections are functionally significant for *H*. *axyridis* and favored by selection.

The effects of symbiotic bacteria are density-dependent. Male-killing correlates with *Spiroplasma* density in species of the genus *Drosophila* [35]. The level of cytoplasmic incompatibility and the level of antiviral protection in *Drosophila* positively correlate with *Wolbachia* density [48, 68–71]. Until now, the mechanisms of density formation for *Spiroplasma* remain unknown. Mechanisms of density formation were studied for *Wolbachia*, and these mechanisms depend both on the characteristics of the host and bacterial genomes. In most cases of multiple *Wolbachia* infections, the density of each strain is under independent host control. The strains do not compete with each other, except for the only known case today, when one *Wolbachia* strain was shown to be suppressed by another strain during co-infection [65, 67, 68, 72–76]. Bacterial density may be under the control of bacterial genes. The density of *Wolbachia* strain *w*MelPop depends on the number of copies of a special operon named Octomom [48, 77]. Ambient temperature and the host diet also influence the bacterial density [75, 78, 79]. The density of symbiotic bacteria is consistently increased during embryonic and larval development [35]. In this study, we showed that two parameters—infections type (mono- or multiple infection) and *Spiroplasma* density are correlated. Molecular mechanisms of genetic control of the *Spiroplasma* density, as well as the biological consequences of single and multiple infections, remain unclear. It can be assumed that the increased density of *Spiroplasma* in beetles infected with several strains can increase the stability of the infection, reducing the likelihood of spontaneous loss of *Spiroplasma*, which explains the wide spread of multiple infection in *H*. *axyridis*. Quite possible *Spiroplasma* strains can interact according to the principle of complementarity. Our data can be the basis for further experimental study of the genetic control of *Spiroplasma* density in *H*. *axyridis*.

The diversity of strains seems to provide *Spiroplasma* with a variety of manipulations with the reproductive strategy of the host. Cytological studies of *Spiroplasma* revealed two scenarios of interactions with *H*. *axyridis* embryos. In the first case, embryo development stops at the stage of yellow eggs, and there are four critical development points, after which embryos die. In the second scenario, embryos died at the stage of gray eggs immediately before hatching [80]. We suggest that such differences can be the result of competition between two *Spiroplasma* strains with different and density dependent effects. Therefore, it is of considerable interest to investigate associations between the type of infection (single or multiple), and the degree of manifestation of male-killing and probably cytoplasmic incompatibility, which has not been shown in *H*. *axyridis*.

## Supporting information

S1 Fig Map of H.axyridis sampling locations. (TIF)
