# Correction: *Spiroplasma* infection in *Harmonia axyridis* - Diversity and multiple infection

**DOI:** 10.1371/journal.pone.0202444

**Published:** 2018-08-09

**Authors:** The PLOS ONE Staff

There are errors in the Funding section. The publisher apologizes for the errors. The correct funding information is as follows: The study of *Spiroplasma* in western group of populations of *H*.*axyridis* was supported by the Russian Foundation for Basic Research, 15-04-07466_A (IG). The study of *Spiroplasma* in eastern group of populations of *H*.*axyridis*—the source of the global invasion, was supported by the Russian Science Foundation, 16-16-00079 (IZ). The funders had no role in study design, data collection and analysis, decision to publish, or preparation of the manuscript.
