# Supplementary figures and images for: Spiroplasma infection in Harmonia axyridis - Diversity and multiple infection
Source: PLoS One. 2018 May 29;13(5):e0198190. doi: 10.1371/journal.pone.0198190 (PMC5973594; doi:10.1371/journal.pone.0198190)

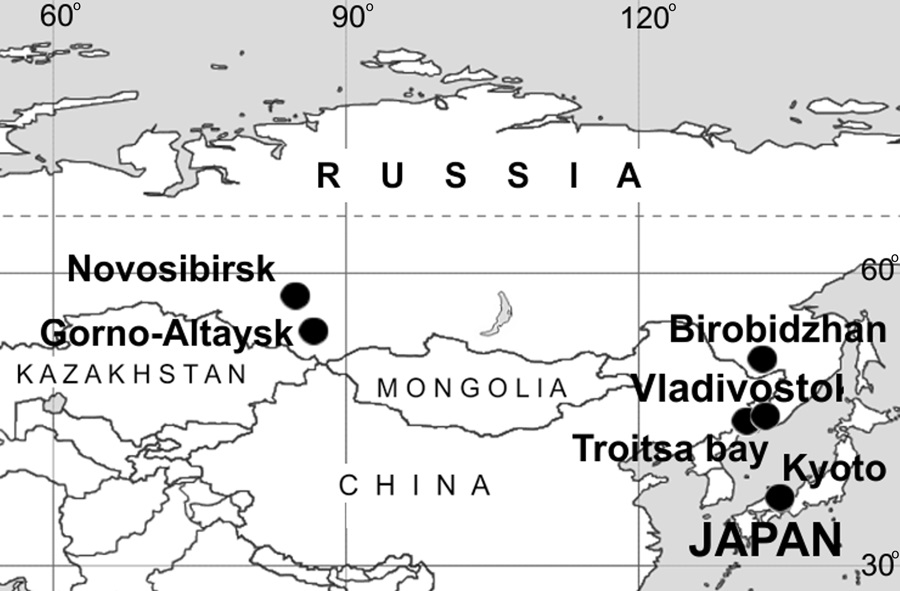

Supplement: S1 Fig — (TIF) [file pone.0198190.s001.tif]
